# Impact of Preoperative Magnetic Resonance Imaging on Surgical Outcomes in Women with Invasive Breast Cancer: A Systematic Review and Meta-Analysis

**DOI:** 10.1155/2022/6440952

**Published:** 2022-08-25

**Authors:** Li Li, Qinghong Zhang, Chunrui Qian, Huien Lin

**Affiliations:** ^1^Department of Medical Imaging, Haikou Maternal and Child Health Hospital, Haikou 570203, China; ^2^Department of Breast Surgery, Haikou Maternal and Child Health Hospital, Haikou 570203, China; ^3^Department of Radiology, Haikou Hospital of Traditional Chinese Medicine, Haikou 570216, China

## Abstract

**Background:**

Currently, whether magnetic resonance imaging (MRI) should be routinely applied to patients with breast cancer before surgery remains controversial. A pooled analysis of the association between preoperative MRI and surgical outcomes in female patients with newly diagnosed invasive breast cancer was conducted to provide evidence-based medicine for clinical practice.

**Methods:**

Three independent researchers searched the following databases: PubMed, Medline, Embase, Ovid, Cochrane Library, and Web of Science from inception to April 2022. Literature was included and excluded according to Cochrane's principles. The basic information from eligible documents was extracted. Systematic evaluation and meta-analysis were performed, and the odds ratio (OR) was analyzed by the random-effect model. The quality of the literature was assessed using the modified Jadad scale and the Newcastle-Ottawa (NOS) mean scale.

**Results:**

A total of 19 studies were included, including 4 randomized controlled trials and 15 observational comparative studies. Among them, most studies were not limited to a specific pathological type, with the exception of 3 that were limited to invasive lobular carcinoma. The results showed that preoperative MRI examination would significantly reduce the reoperation rate (OR = 0.77, *P*=0.02) and increase the mastectomy rate (OR = 1.36, *P*=0.001). In comparison, preoperative MRI did not significantly affect the rate of secondary mastectomy (OR = 0.77, *P*=0.02), the rate of positive margin (OR = 1.08, *P*=0.66), the rate of mastectomy (OR = 1.00, *P* < 0.05), and reoperations (OR = 0.65, *P*=0.19) in the subgroup analysis of patients with invasive lobular carcinoma.

**Conclusion:**

Available evidence suggests that preoperative MRI examination increases the rate of mastectomy and reduces the rate of reoperations. The results indicate that preoperative MRI examination has the potential to benefit patients with breast cancer, but more high-quality studies are needed for confirmation.

## 1. Introduction

Currently, whether magnetic resonance imaging (MRI) should be routinely applied to patients with breast cancer before surgery remains controversial. Several earlier clinical evidence-based studies did not find evidence that supported routine preoperative MRI to be beneficial for surgical treatment. Nonetheless, these studies also acknowledged that preoperative MRI could detect some other diseases that failed to be detected by other conventional preoperative imaging examinations. However, current data from various studies failed to reach a unified clinical opinion [1–5]. A prior study indicated that the high sensitivity of preoperative MRI enabled the finding of coexisting diseases, such as breast hyperplasia or fibroma, in patients with breast cancer [5]. The results from several randomized controlled trials (RCTs) and observational studies were inconsistent and confusing [6–9]. Meanwhile, no conclusive evidence indicated that MRI improved surgical treatment or led to more extended surgery [10–12]. Thus, the impact of preoperative MRI on patients with breast cancer is constantly changing and contradictory. Although some meta-analysis studies focused on the detection ability of MRI [13] and the increase in mastectomy, they failed to incorporate other surgical outcomes [14]. Since the meta-analysis results are unclear, the current recommendations and guidelines on whether to use MRI in patients with newly diagnosed breast cancer before surgery are also different [15, 16].

This paper summarized and analyzed the relationship between preoperative MRI and surgical treatment in newly diagnosed female invasive breast cancer patients. This paper included more updated clinical studies to ensure that the analysis results included all the current evidence. Meanwhile, the subgroup analysis was performed to obtain more accurate results and avoid bias.

## 2. Materials and Methods

### 2.1. Literature Retrieval Strategy and Inclusion/Exclusion Criteria

The literature published before April 2022 was searched following the principle of Cochrane. Three independent researchers searched the following databases respectively: PubMed, Medline, Embase, Ovid, Cochrane Library, and ISI Web of Science, with the search formula of “breast neoplasms”[All Fields] OR “breast cancer” [All Fields])) AND (“magnetic resonance”[Title] OR “MRI”[Title]) AND (“pre-operative”[Title] OR “pre”[Title]). Disputes arising from retrieval were resolved through negotiation and discussion.

The inclusion criteria were 1. controlled trial research; 2. english publications; 3. study subjects were females with invasive breast cancer receiving surgical treatment; 4. preoperative breast MRI examination was performed; 5. detailed surgical treatment data available, including data about primary surgery and reoperations; 6. curative surgical modalities performed, including excision surgery and breast-conserving surgery (BCS).

The exclusion criteria were 1. uncontrolled pilot studies, such as those that only evaluated MRI in breast cancer patients without a control group; 2. noncurative surgeries performed, such as cosmetic or palliative surgery; 3. studies without preoperative MRI or surgical data; 4. non-English articles; 5. nonoriginal articles, such as case reports, reviews, and correspondence; 6. animal studies; 7. study quality, as evaluated by the modified Jadad scale score or the NOS (Newcastle-Ottawa scale) score, was ≥4 for RCT or ≥4 for observational studies.

### 2.2. Research Endpoints and Literature Data Extraction

The primary endpoint of this meta-analysis was the mastectomy rate in women with invasive breast cancer. Secondary endpoints included whether primary breast-conserving surgery patients underwent secondary surgery for mastectomy, positive margins during breast-conserving surgery, reoperations, and prophylactic mastectomy.

Two researchers extracted the basic information from eligible literature, followed by cross-checking by a third researcher. The extracted data included literature characteristics (author, year of publication), patient characteristics (number, tumor size, pathological type), and evaluation of surgical outcomes. The following surgical outcomes were studied, including the number of people whose first operation was mastectomy or BCS, the number of patients with BCS at the first operation and positive margin, the number of patients who underwent BCS for the first time and underwent a secondary mastectomy, and the total number of patients undergoing a secondary mastectomy.

### 2.3. Document Quality Evaluation

Risk of bias plots were prepared using the Risk of Bias analysis tool in Review Manager software following the analysis guidelines provided by The Cochrane Library. Risk of bias analysis includes random sequence generation, allocation concealment, participant and subject blinding, outcome assessment blinding, completeness of outcomes, outcome reporting bias, and other biases. The risk of bias includes three levels, namely low, high, and unclear, and the results are marked with three color blocks of red, green, and yellow. The modified Jadad scale and the NOS scale were used for the evaluation of RCT and observational study, respectively. The modified Jadad scale was divided into four parts, including random sequence generation (2 points), randomized hiding (2 points), blind method (2 points), withdrawal, and dropout (1 point), for a total of 7 points. A score of 1–3 points or 4–7 points signified low-quality and high-quality research, respectively. The observational study was evaluated with the NOS scoring table that included three parts with a total of 9 points, including the selection of subjects in the case and control groups (4 points), the comparability of cases and controls (2 points), and the measurement of exposure factors (3 points). Low-quality research scores 1–6 points, and high-quality research scores 7–9 points.

### 2.4. Statistical Analysis

The Review Manager software (version 5.4 of the Nordic Cochrane Centre, Copenhagen, Denmark) was used to conduct a random meta-analysis and establish a forest map for comparison of the total effect between the MRI examination group and the control group. Due to the heterogeneity within and between studies, the standard deviation, 95% confidence interval (CI), and *P* value analysis were carried out. The data used in each study were different, but all studies have reported the corresponding odds ratio (OR), so the final summary data were summarized into the forest map in the form of OR with 95% CI. The random-effect model was used in the presence of significant heterogeneity. The studies with clinical homogeneity were divided into subgroups to analyze the specific effects of MRI examination in patients with different pathological types of breast cancer. The chi-square test was used for the heterogeneity test. Two-sided *P* < 0.05 denoted statistical significance.

## 3. Results

### 3.1. Characteristics and Quality Evaluation of Included Articles

**Figure 1:**
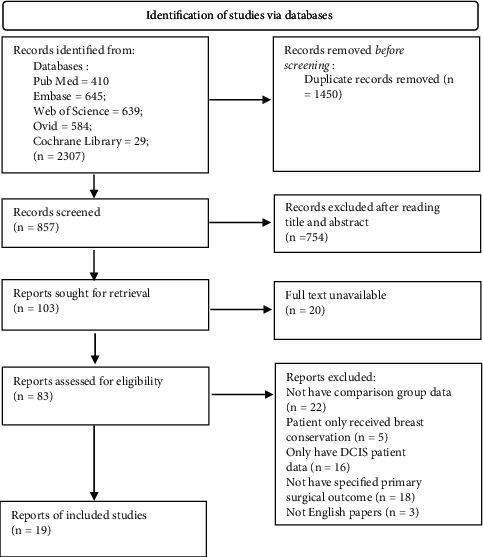
Document screening and exclusion process.

A total of 2307 studies were retrieved from database. After screening (Figure 1), 19 studies [6, 7, 17–32], including 4 RCTs and 15 observational comparative studies, were included. Among the 19 publications, 16 focused on newly diagnosed breast cancer patients that were not limited to pathological types. The other 3 studies only focused on patients with breast invasive lobular carcinoma (ILC). The majority of literature included in the study excluded patients after neoadjuvant therapy, with the exception of only 1 study [33].

**Figure 2:**
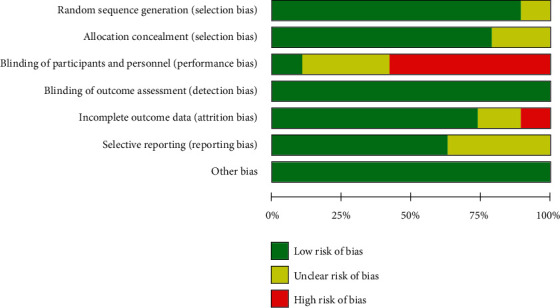
Summary of risk of bias for included studies.

**Figure 3:**
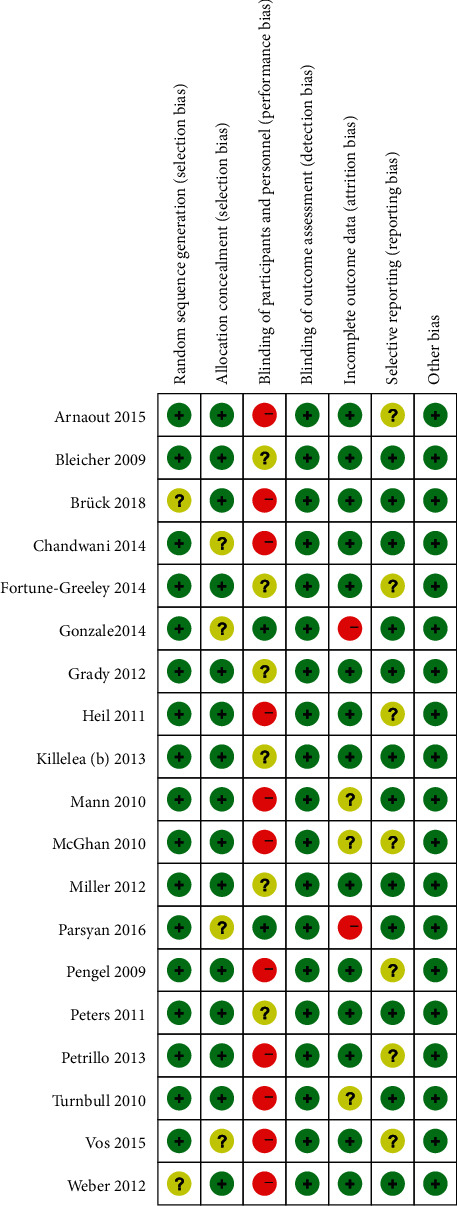
Risk of bias summary.

Figures 2 and 3 are a summary of the risk of bias and a bar chart for the detailed analysis of the risk of bias for each included literature, respectively. In general, most studies used randomization protocols and reported results that were low-risk when completed. Most studies have certain defects in the blinding of participants and subjects, and most of them are single-blind studies with high risks. The detailed characteristics of each study are shown in Table 1. The total number of subjects included was 86701, of which 15587 patients received preoperative MRI. In most studies, the median or average age of patients was close, with some heterogeneity. The median or average age of patients undergoing MRI in the included studies was lower than that of patients who did not. In this study, the modified Jadad scale score and the NOS score were 4–5 and 7–8, respectively.

### 3.2. Effect of the Preoperative MRI Mastectomy Rate

**Figure 4:**
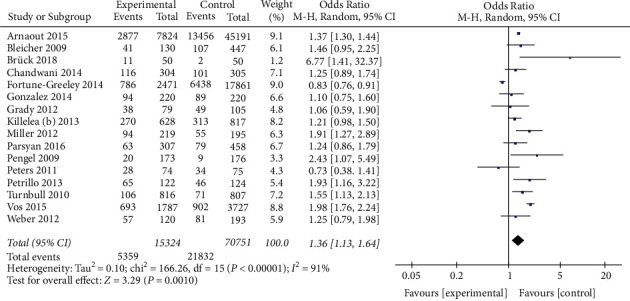
Effect of preoperative MRI on the rate of mastectomy in patients with breast cancer.

A total of 86075 patients from 16 studies were included in the analysis to analyze the effect of preoperative MRI on the rate of mastectomy. The data (Figure 4) analyzed by the random-effect model (*I*^*2*^ = 91%) showed that preoperative MRI examination in patients with breast cancer was associated with an increased mastectomy rate (OR = 1.36, 95% CI = 1.13, 1.64, *Z* = 3.29, *P*=0.001).

### 3.3. Effect  of MRI on the Reoperation Rate

**Figure 5:**
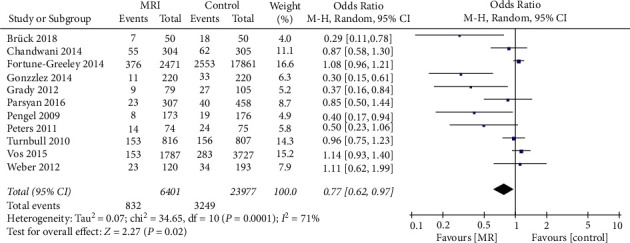
Effect of preoperative MRI examination on the reoperation rate in patients with breast cancer.

A total of 11 pieces of literature with 30378 patients were pooled to analyze the reoperation rate after preoperative MRI examination. Significant interstudy heterogeneity was noted (*I*^*2*^ = 71%). The data, as presented in Figure 5, showed that patients with breast cancer who received an MRI examination before the operation would significantly reduce the reoperation rate (OR = 0.77, 95% CI = 0.62, 0.97, *Z* = 2.27, *P*=0.02).

### 3.4. Effect of Preoperative MRI Examination on the Rate of Primary BCS and Secondary Mastectomy

**Figure 6:**
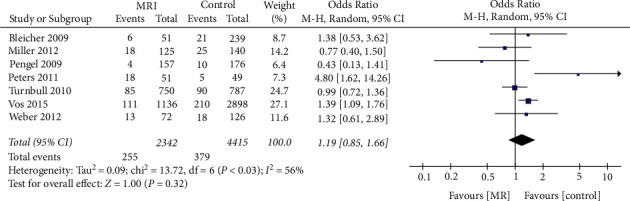
Effect of preoperative MRI examination on the rate of primary breast-conserving surgery and secondary mastectomy in patients with breast cancer.

To analyze the rate of secondary mastectomy following BCS, data from 7 studies that included 6757 patients were merged. We found that the preoperative MRI examination did not significantly affect the rate of primary BCS and secondary mastectomy (Figure 6) between the two groups (OR = 1.19, 95% CI = 0.85, 1.66, *Z* = 1.00, *P*=0.32, Figure 6). Significant heterogeneity among studies was observed (*I*^*2*^ = 56%).

### 3.5. The  Effect of MRI Examination on the Positive Margin Rate of Patients with Breast Cancer Undergoing First-Time BCS

**Figure 7:**
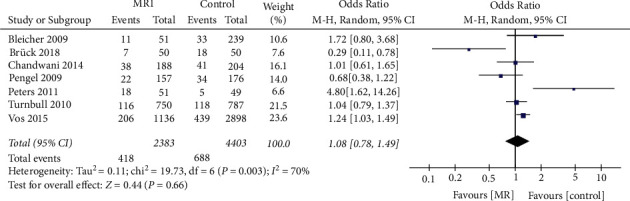
The influence of MRI examination before the operation on the positive rate of incisional margin of patients with breast cancer undergoing breast-conserving surgery for the first time.

The impact of preoperative MRI examination on the positive margin rate for patients undergoing BCS was evaluated in 6786 patients from 7 studies. Random-effect model analysis suggested that preoperative MRI did not significantly affect the rate of positive margin for those receiving BCS (OR = 1.08, 95% CI = 0.78, 1.49, *Z* = 0.44, *P*=0.66, Figure 7). *I*^*2*^ = 70% indicated significant heterogeneity among studies.

### 3.6. Effect of Preoperative MRI on the Mastectomy Rate in Patients with Breast ICL

**Figure 8:**
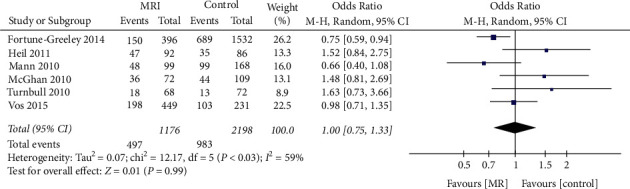
Effect of preoperative MRI examination on the mastectomy rate in patients with breast cancer in the ICL subgroup.

Preoperative MRI was performed for ICL in 6 publications that included 3374 patients. Subgroup analysis (Figure 8) found no significant difference regarding the mastectomy rate for ICL patients receiving preoperative MRI or not (OR = 1.00, 95% CI = 0.75, 1.33, *Z* = 0.01, *P*=0.99, Figure 8).

There was significant heterogeneity among studies (*I*^*2*^ = 59%).

### 3.7. Effect of Preoperative MRI Examination on the Reoperation Rate in the ICL Subgroup

**Figure 9:**
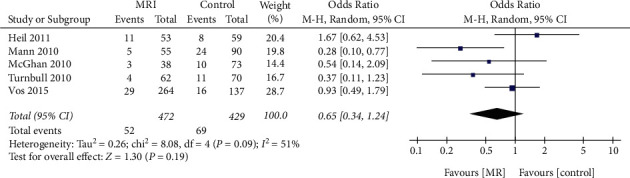
Effect of preoperative MRI examination on the reoperation rate in patients with breast invasive lobular carcinoma.

Subgroup analysis of 901 patients in 5 articles that reported the reoperation rate in ICL showed no significant difference in terms of the reoperation rate in ICL patients with preoperative MRI examination or not (OR = 0.65, 95% CI = 0.34, 1.24, *Z* = 1.30, *P*=0.19, Figure 9). Significant interstudy heterogeneity was observed (*I*^*2*^ = 51%).

## 4. Discussion

Abundant studies have discussed the critical role of preoperative MRI in clinical practice for patients with breast cancer, such as screening for metastasis, monitoring the effect of neoadjuvant therapy, and even assisting in predicting long-term recovery [34–38]. In this study, we comprehensively evaluated the relationship between preoperative MRI and surgical outcomes in patients with breast cancer, thus providing the latest evidence. Previous studies focused on invasive breast cancer and preoperative MRI [39], so studies involving simple DCIS were excluded from the study. A total of 19 studies were included in this analysis which included 86701 subjects. The final results showed that preoperative MRI examination would significantly reduce the reoperation rate (OR = 0.77, 95% CI = 0.62, 0.97, *Z* = 2.27, *P*=0.02) and increase the mastectomy rate (OR = 1.36, 95% CI = 1.13, 1.64, *Z* = 3.29, *P*=0.001). Preoperative MRI examination leads to an increased probability of mastectomy while reducing the recurrence rate and increasing the long-term survival rate in the meantime. However, the clinical effect of preoperative MRI on the recurrence rate and long-term survival rate remains contradictory, with some reporting a favorable effect whereas others did not note this association, which might be related to the characteristics of the patients [8, 40–43]. However, several latest clinical studies indicated a beneficiary role of constantly innovative qualitative and quantitative analysis of MRI. Although novel MRI is still in its infancy, it is of great clinical significance [44, 45]. Consistently, preoperative MRI examination may be beneficial in improving the long-term survival rate, despite the increased probability of mastectomy. However, this argument is still controversial and needs to be confirmed by additional follow-up data. This is also the difference between this study and other previous analyses. This study found that preoperative MRI has potential benefits for patients.

This study also analyzed other secondary outcomes. The results showed that breast cancer patients who received preoperative MRI examinations were associated with a reduced reoperation rate. Other secondary outcomes were not affected by preoperative MRI. The use of preoperative MRI increased the rate of mastectomy, which was associated with an extended surgical field that obviated reoperations. Still, at the same time, the decline in the rate of BCS might reduce the benefit. More research with follow-up data is needed to determine the real benefits.

A host of early clinical evidence indicated that preoperative MRI did not benefit patients with newly diagnosed breast cancer. However, preoperative MRI is still widely accepted and used in the clinic [35, 46]. At present, the investigation and study of surgeons treating breast cancer patients during 2013–2015 found that 60% of doctors recommended MRI examination in patients aged 45 or below, and 26% of surgeons used MRI to screen for early breast cancer [47]. In addition, 41% of surgeons believed that preoperative MRI may not increase the possibility of mastectomy, and 29% deemed that MRI reduced the likelihood of secondary surgery for patients undergoing BCS [47]. These investigations indicated that clinicians had insufficient understanding of the role of preoperative MRI. Therefore, our study aims to provide updated and more comprehensive clinical evidence to help resolve clinical difficulties.

We still analyzed the utility of preoperative MRI in the ICL subgroup despite limited data. General analysis showed that preoperative MRI did not affect the probability of mastectomy and secondary surgery rate in patients with breast ILC, which was also consistent with other clinical studies [46]. A considerable proportion of surgeons recommended MRI evaluation of ICL patients before the operation because B-ultrasound or mammography easily underestimated the invasive range of ICL [47]. However, the metadata of this study showed that female patients with ICL could not benefit from preoperative MRI, which was contradictory to some expert consensus or guidelines. Due to the limited number and considerable heterogeneity of included studies, this result should be interpreted carefully.

Compared with previous studies such as Houssami 2013 and Houssami 2017 [14, 46], this study is superior in terms of the breadth and quality of included literature, and the inclusion of updated studies. Furthermore, we found that receiving MRI before surgery increased the rate of mastectomy in patients with new breast cancer but reduced the rate of secondary surgery, which is different from those reported previously. This is critical in the sense that preoperative MRI may benefit breast cancer patients and thus guide the clinical use of preoperative MRI. The conclusions of prior studies are biased against the application of preoperative MRI.

This study also suffers from several limitations. Since many of the included studies were observational with insufficient randomization, bias and confounding factors could be eliminated, and the heterogeneity between studies was significant. In addition, the age and tumor size in each study varied greatly, which was also one of the reasons for the high heterogeneity. Therefore, this paper adopted improved Jadad and NOS scores to include high-quality studies and reduce the heterogeneity of research.

In conclusion, this study compared the surgical outcomes in patients with newly diagnosed breast cancer who received a preoperative MRI examination with those who did not. The results showed that receiving a preoperative MRI examination would increase the mastectomy rate and reduce the reoperation rate. Therefore, preoperative MRI examination may benefit patients with breast cancer. The results of this study may have an impact on the formulation of guidelines, clinical practice, and application of health and financial resources. More research data are needed to conclusively determine the utility of preoperative MRI for breast cancer.

## Figures and Tables

**Table 1:** Characteristics and quality scores of included publications.

Study	Study type	Number of patients		Age (years)		Tumor size (mm)		Proportion screen-detected		Proportion ILC		Grade
		MRI	Control	MRI	Control	MRI	Control	MRI	Control	MRI	Control	
Brück [7]	RCT	50	50	61	61	15.0	13.0	68%	52%	0%	0%	5
Turnbull [19]	RCT	816	807	57.0	57.0	15^∗^	15^∗^	52%	52%	9.0%	10.0%	4
Peters [18]	RCT	74	75	55.1	56.1	15	15.1	100%^#^	100%^#^	NR	NR	4
Gonzalez [33]	RCT	220	220	46	46	NR	NR	37.7%	37.7%	NR	NR	5
Pengel [30]	Comparative	173	176	56.8	59.2	16.9	15.7	30%	39%	16.4%	14.4%	7
Bleicher [31]	Comparative	130	447	52.5	59	21	21	44%	45%	15.4%	11.2%	7
Miller [26]	Comparative	219	195	51.0	56.0	NR^±^	NR^±^	53%	60%	12.0%	7.0%	8
Weber [25]	Comparative	120	193	53.6	59.5	20.2	17.2	NR	NR	8.3%	5.2%	7
Grady [32]	Comparative	79	105	63	64	NR	NR	NR	NR	14.0%	9.0%	7
Petrillo [23]	Comparative	122	124	34.8	34.7	NR	NR	NR	NR	11.5%	8.9%	8
Killelea [24]	Comparative	628	817	53	60	16	15	NR	NR	12%	11%	8
Fortune-Greeley [22]	Comparative	2471	17861	NR	NR	NR	NR	NR	NR	16.0%	8.6%	8
Chandwani [17]	Comparative	304	305	NR	NR	NR	NR	NR	NR	11.5%	9.2%	7
Arnaout [6]	Comparative	7824	45191	NR	NR	NR	NR	NR	NR	10%	5.9%	7
Vos [21]	Comparative	1787	3727	58	63	NR	NR	41.3%	42.6%	25.1%	6.2%	7
Parsyan [20]	Comparative	307	458	55.3	66.3	17.6	17.7	NR	NR	13.0%	10.50%	7
Mann [29]	Comparative	99	168	57.0	60.0	24	23	NR	NR	100%	100%	7
McGhan [28]	Comparative	72	109	62.7	68.2	24.4	21.3	NR	NR	100%	100%	8
Heil [27]	Comparative	92	86	57.8	63.6	NR	NR	NR	NR	100%	100%	8

RCT: randomized controlled trial; comparative: observational comparative study; age use of median or mean; ILC: invasive lobular cancer; NR: not reported; grade: RCT uses the M-Jadad scale; comparative uses the NOS scale.

## Data Availability

The data used and analyzed during the current study are available from the corresponding author.
